# Health and lifestyle in the Iron Age Italian community of Pontecagnano (Campania, Italy, 7th-6th century BCE)

**DOI:** 10.1371/journal.pone.0338448

**Published:** 2026-01-14

**Authors:** Roberto Germano, Owen Alexander Higgins, Emanuela Cristiani, Alessia Galbusera, Carmen Esposito, Dulce Neves, Carmine Pellegrino, Alessandra Sperduti, Giorgio Manzi, Luca Bondioli, Alessia Nava

**Affiliations:** 1 Department of Environmental Biology, Sapienza University of Rome, Rome, Italy; 2 Department of Odontostomatological and Maxillofacial Sciences, Sapienza University of Rome, Rome, Italy; 3 Department of Cultural Heritage, University of Bologna, Ravenna, Italy; 4 Research Centre for Anthropology and Health (CIAS), Department of Life Sciences, University of Coimbra, Coimbra, Portugal; 5 Department of Cultural Heritage Sciences, University of Salerno, Salerno, Italy; 6 Bioarchaeology Service, Museum of Civilizations, Rome, Italy; 7 Asia Africa and Mediterranean Department, University “L’Orientale” of Naples, Italy; 8 Institute of Geological Sciences, Polish Academy of Sciences, Kraków, Poland; University of South Florida, ITALY

## Abstract

This study investigates health, dental development, diet, and human-environment interactions in individuals buried in the necropolises of Pontecagnano (Campania, Italy, 7th-6th century BCE), using an integrated approach merging dental histomorphometry and calculus micro-residue analysis. The sample consists of 30 permanent teeth (canines, first and second molars) from 10 individuals. Histomorphometric analysis of dental thin sections allowed the estimation of crown formation times, initial cusp formation, crown completion, and enamel extension rates. The prevalence of Accentuated Lines, marking physiological stress events, was analyzed chronologically across tooth classes. Dental calculus analysis was performed on five individuals, identifying plant micro-remains and fungal spores. Crown formation times varied by tooth class, with canines forming the longest (mean = 1,977 ± 295 days), followed by second molars (mean = 1,176 ± 179 days) and first molars (mean = 1,094 ± 154 days). Initial cusp formation values, estimated through chronological overlap between teeth, allowed for a more accurate reconstruction of crown completion timing. Accentuated Lines prevalence peaked at 12 and 44 months, likely reflecting early childhood dietary transitions and the differential recording of stress events across different crown regions. Calculus analysis identified starch granules from cereals (Triticeae) and legumes (Fabaceae), fungal spores (*Saccharomyces*), and plant fibers, indicating diverse dietary practices, food processing, and extra-masticatory activities. This interdisciplinary approach reinforces the validity of combining histomorphometric and micro-residue analyses to reconstruct childhood health, adult diet, and lifestyle. Our findings align with previous research while emphasizing population-specific variations. This study enhances understanding of Iron Age biocultural adaptations, offering insights into developmental and dietary behaviors in this ancient Italian community.

## Introduction

The study of teeth has emerged as a crucial approach in bioarchaeology, combining methods such as dental histology and micro-residue analysis to reconstruct individual life histories [1,2]. Teeth, as reliable biological archives, not only record the incremental formation of dental mineralized tissues – offering unique insights into childhood growth and health [3,4] – but can also preserve dental calculus, which serves as a repository for micro‐inclusions related to diet and even occupational or para-masticatory activities later in life [5,6]. By integrating dental histomorphometry, with a particular focus on dental enamel, and calculus analysis, it is possible to obtain a detailed picture of both biological life histories during infancy, childhood, and adult lifestyles, enhancing our understanding of past populations adaptive capacity and cultural behaviors [5–10].

Teeth are excellent archives due to the incremental formation of their tissues and resistance to diagenetic and post-depositional alteration [11–15]. They act as individual biological archives providing information about responses to living conditions and environmental stresses during infancy and childhood [1,3,16]. Dental enamel, once formed, retains its original structure without undergoing physiological remodeling [17]. Its rhythmic formation produces microscopic physiological incremental lines regularly repeated in the dental crown, allowing the tracking of information and the ability to “count” the days in an individual’s life [3,11,18–20].

Dental histology of deciduous and permanent teeth is a valuable tool for obtaining information on the development and health status of individuals from before birth to the end of dental development [3,4], helping to partially address challenges associated with the Osteological Paradox [21,22], particularly concerning selective mortality. Histomorphometric analysis of dental crowns allows the study of formation times, the chronology of non-specific physiological stresses observable as Accentuated Lines (ALs), and individual life histories [1,3]. Stress markers appear simultaneously in the crowns of all teeth, forming at the same time, ultimately enabling a pseudo-longitudinal study of childhood by using multiple teeth to obtain a more comprehensive view of the individual’s life history [3,19].

While dental histology primarily provides insights into infancy, childhood, and adolescence, which are the windows of dental crown formation, the presence of dental calculus (mineralized plaque) extends the temporal range of study into adulthood. Dental calculus – formed through the mineralization of plaque – preserves a wide array of information related to diet, health, and other aspects of daily life. Oral plaque can calcify already within two weeks, accreting episodically after tooth eruption. If not mechanically removed, this record can span months to years prior to death [5]. For this reason, it represents a long-term reservoir of dietary/oral/host biomolecules and other debris resulting from dietary habits, accidental ingestion/inhalation, and the use of the mouth as a “third hand” in para-masticatory activities [6,9,23,24].

This research explores the timing of dental development, infancy and childhood health status and adult diet of the inhumed individuals from the Pontecagnano necropolises (Campania, Italy, 7th-6th century BCE) [25], one of the largest pre-Roman site in Southern Italy, using histomorphometry and microscopy analysis of dental calculus from permanent teeth. The site of Pontecagnano offers a unique opportunity to unravel the bio-cultural responses to emerging social and environmental challenges during the Orientalizing period (720–580 BCE) when borders, political balance, and networks all over the peninsula were entirely redesigned [26]. These transformations were accompanied by intensified trade and cultural contacts with the Greek world, increasing social stratification, and shifts in settlement organization [27–29]. Such dynamics may have influenced health and lifestyle through possible exposure to new pathogens via mobility and exchange networks, and unequal access to resources linked to emerging of new social hierarchies, while broader economic and environmental pressures could also have affected living conditions. In this context, studying individual life histories during childhood – when individuals are most vulnerable and entirely dependent on the care of others – provides insights into how a population responds to these bio-cultural challenges and changes [7,8,30], enhancing our understanding of the Iron Age community of Pontecagnano and their environmental and social context.

### Dental enamel microstructures

Enamel, composed of approximately 95–97% inorganic materials (primarily hydroxyapatite) and 3–5% organic materials and water [31,32], develops in incremental layers starting from the enamel-dentine junction (EDJ) towards the outer surface of the tooth. This rhythmic process results in the formation of microscopically visible growth markers. These include cross-striations, representing daily growth increments, and Retzius lines, which form at longer intervals, usually on a near-weekly basis [12,33,34].

Cross-striations are visible along the enamel prisms when observed under transmitted light microscopy. They appear as alternating light and dark bands, representing the daily secretion rate of ameloblasts [12,31]. These striations result from the regular variation in the rate of enamel matrix secretion, reflecting the circadian rhythm of ameloblast activity [34,35]. The cross-striations serve as fundamental units for constructing a chronological timeline of enamel formation [19,36].

Retzius lines (or striae of Retzius) are incremental lines that, in longitudinal cross sections, appear as sub-parallel lines representing the depositional fronts of the ameloblasts at different times of development [12]. Accentuated Lines (ALs) are stress-affected Retzius lines that, due to temporary disruptions of the normal ameloblast activity, appear darker and more pronounced compared to regular Retzius lines [37,38]. It is not possible to associate an AL with a specific stress, as ALs are non-specific physiological stress markers that can form in response to various factors, including malnutrition, infectious diseases, and psychological stress [12,38]. The first notable AL observed in teeth is usually the Neonatal Line (NL), which marks the birth event and separates prenatal from postnatal enamel [39–41]. However, several studies indicate it is possible to find ALs in prenatal enamel as well [30,42–48]. The presence of the NL and other ALs provides critical information for registering life histories across teeth of the same individual and identifying periods of stress during enamel formation [12].

By registering ALs across multiple teeth, it is possible to construct a cohesive developmental timeline, offering a longitudinal perspective of childhood growth and stress events [3,49]. This method allows to overcome limitations associated with mortality samples [21,22], by focusing on developmental periods long before an individual’s death. Overall, the study of dental enamel microstructures allows for the reconstruction of the timing and duration of enamel formation, the identification of periods of physiological stress, and a deeper understanding of past human experiences [12,33,38,50].

### Dental calculus

The formation of dental calculus, or tartar, results from the interaction between salivary components and oral bacteria, leading to a calcified biofilm on tooth surfaces if not regularly removed [5,51,52]. The rate of calculus accumulation varies among individuals and is influenced by several factors such as diet, oral pH and hygiene, among others [53]. During the mineralization, dental calculus can entrap a wide variety of microscopic particles and compounds present in the oral cavity and the living environment, ranging from plant micro-remains (e.g., starch granules, phytoliths, wood fragments, bast fibers), to animal-derived residues (e.g., feather fragments, fish scales, collagen fibers), as well as fungi, diatoms, mineral particles, and microorganisms. Optical analyses of dental calculus under cross-polarized transmitted light allow the identification of micro-remains associated with food consumption and daily habits, as well as other non-dietary practices [6,9,54,55]. In addition to morphologically identifiable debris, dental calculus may preserve chemical compounds and biomolecules, such as secondary metabolites, bacterial and host DNA, which can be analyzed through biochemical approaches, including proteomics and metagenomics approaches [56]. Its paleopathological relevance has been increasingly recognized, as dental calculus can preserve microbial signatures and biochemical markers indicative of oral and systemic health conditions [57,58]. Thanks to its heterogeneous composition, dental calculus represents a powerful and long-term reservoir for reconstructing not only ancient diets, but also health, occupational habits, and biocultural practices in both ancient and modern human populations and extinct hominins species such as Neanderthals [5,6,10,59].

Although calculus analysis requires caution due to complex formation processes, when combined with isotopic, archaeobotanical and functional evidence, it provides valuable insights into individual and population-level lifeways [5,60]. More recently, dental calculus has also been explored within the framework of ancient medicine. Micro-remains and biochemical markers embedded in this material have revealed the use of plants with potential therapeutic properties, suggesting that past societies possessed empirical knowledge of local botanicals and may have engaged in intentional health care practices [59]. These findings underscore the potential of dental calculus to reveal previously inaccessible aspects of ancient medicinal knowledge and cultural approaches to health.

## Materials and methods

### Dental sample

**Fig 1:**
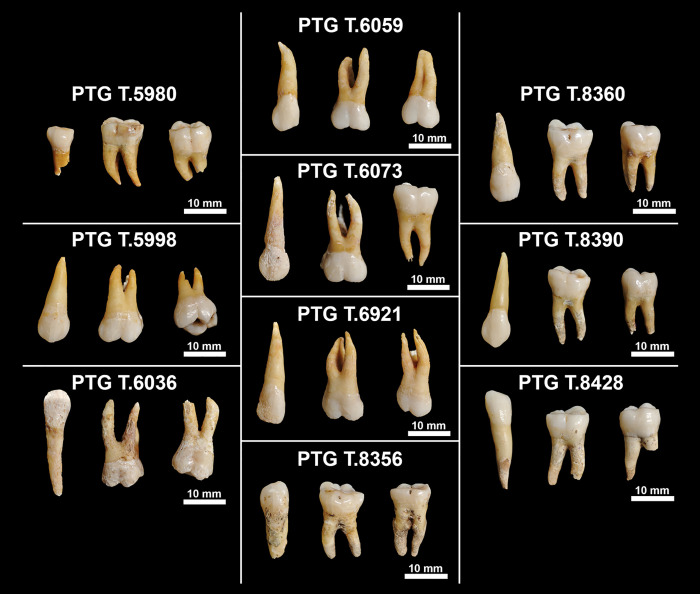
Dental sample. The 30 permanent teeth of the 10 individuals from the Iron Age necropolises of Pontecagnano selected for histological analysis, divided by tombs. All teeth are in buccal view.

The selected dental sample for histological analysis consists of 30 permanent teeth (Fig 1) from 10 individuals from the Proprietà Baldi, Chiancone and Proprietà Gaeta Iron Age necropolises of Pontecagnano (Campania, Italy, 7th-6th century BCE) [25]. The sample size reflects both the strict selection criteria applied to ensure optimal preservation of dental tissues for histological analysis and the ethical need to minimize destructive sampling of archaeological human remains. The individuals were selected based on the wear stage of specific dental classes, ensuring that canines did not exceed wear stage D and molars did not exceed wear stage F, according to Lovejoy’s dental wear scale [61]. Age at death and biological sex of the individuals were independently estimated by two of the authors (AN and AS) following standard anthropological methods [62–64]. Age at death for the selected sample ranges between 16 and 40 years, and both males and females are represented (Table 1).

**Table 1:** Summary of individuals, sampled teeth, and analyses conducted in this study.

ID sample	ID tooth	Sex	Age at death (years)	Analyses performed
	LLP4			
PTG T.5980	LLM1	?	25-30	Histology
	LLM2			
	ULC			
PTG T.5998	ULM1	M	35-45	Histology and dental calculus microremains
	ULM2			
	LLC			
PTG T.6036	ULM1	F	20-30	Histology
	URM2			
	URC			
PTG T.6059	URM1	F	20-30?	Histology
	URM2			
	URC			
PTG T.6073	URM1	M	20-30	Histology
	LLM2			
	URC			
PTG T.6921	URM1	?	20-30	Histology
	URM2			
	LLC			
PTG T.8340	LRM1	F	~40	Dental calculus microremains
	LRM2			
	LRC			
PTG T.8356	LLM1	?	~40	Histology and dental calculus microremains
	LRM2			
	ULC			
PTG T.8360	LLM1	M	18-21	Histology and dental calculus microremains
	LLM2			
	URC			
PTG T.8390	LLM1	F?	15-18	Histology
	LLM2			
	LLC			
PTG T.8428	LRM1	M?	20-30	Histology and dental calculus microremains
	LRM2			

The table reports biological sex (M = male, M? = probable male, F = female, F? = probable female,? = undetermined), estimated age at death range (~ = approximate age), and the type of analysis performed.

For each individual, three permanent teeth were sampled: one canine, one first molar, and one second molar. In one case (PTG T.5980), as canines were not available, a fourth premolar was chosen instead. When possible, teeth were selected from the same dental quadrant. These specific classes of teeth were selected for their chronological overlapping during crown formation, enabling a pseudo-longitudinal study of the individuals’ childhood from birth to around 7 years of age [3,49,65].

The dental calculus samples consist of a subset of four individuals from the same samples used for the histological analysis, and one additional individual (PTG T.8340, not used for histological analysis due to excessive wear), for a total of five individuals (Table 1). Only a subset of the original sample was used for these analyses, as not all individuals in the dental sample had mineralized calculus on their teeth. Dental calculus was sampled from all three tooth classes in individuals with it, which was present in mild to moderate amounts [66].

The odontoskeletal collection is kept at the *Museo delle Civiltà di Roma* under the curation of one of the authors (AS). All permits were obtained for the described study by the *Museo delle Civiltà di Roma*, which complied with all relevant regulations.

### Pontecagnano necropolises

**Fig 2:**
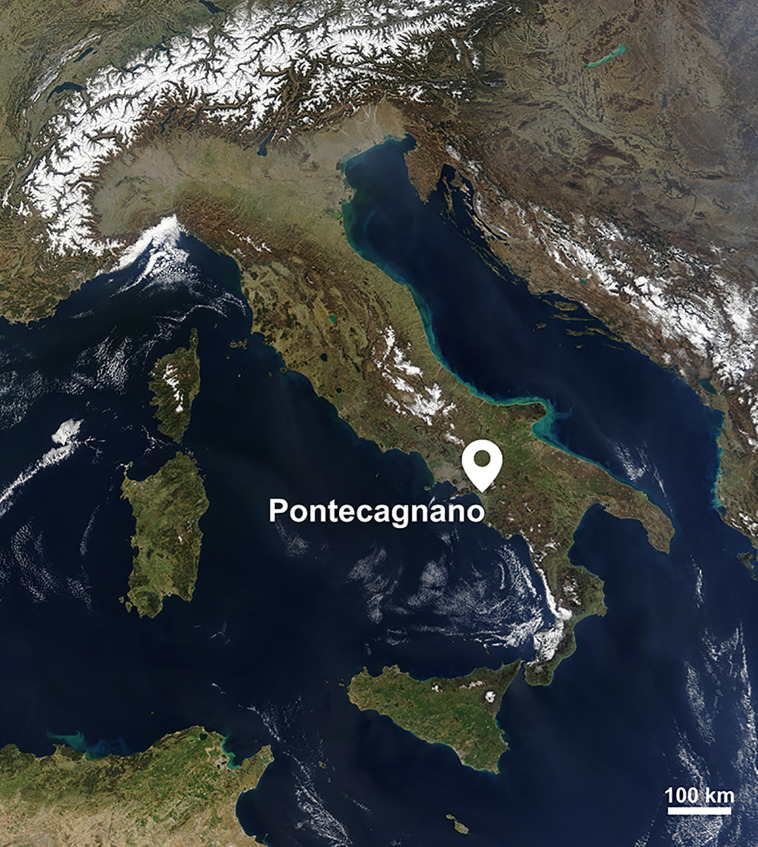
Location of Pontecagnano. Satellite imagery of Italy highlighting the geographical location of Pontecagnano (from NASA Visible Earth project ‒ credits to Jacques Descloitres, MODIS Rapid Response Team, NASA/GSFC).

The site of Pontecagnano (Fig 2), located 8 km southeast of Salerno (Campania, Italy), testifies the Etruscan expansion within Southern Italy [67]. Pontecagnano was founded in the early 9th century BCE by groups of Villanovan culture from southern Etruria [67,68], but see also Peroni [69] for a different interpretation. The site structure and territorial organization closely resemble the protourban centers of Tyrrhenian Etruria. The settlement – which then became the Etruscan city of historical time – was surrounded by numerous necropolises which yielded over ten thousand burials spanning from the 9th to the 3rd century BCE [70].

As in other Iron Age Italian contexts, funerary evidence has been instrumental in reconstructing community developments, owing to the abundance and diversity of material culture and grave goods. The most ancient phases in Pontecagnano are mostly represented by the Villanovan cremation ritual [67]. Subsequently, in the 8th century BCE, inhumations increased [71], but cremations were still frequent. During the Orientalizing period (720−580 BCE) Pontecagnano witnessed a significant economic expansion as well as an increase in cultural contacts with the Greek world [28]. These economic changes are reflected in the necropolises, where strong elite groups were established, represented by a different funerary costume. Inhumation became the prevalent ritual, with only a few cremations representing prominent individuals [29]. The character of the grave goods and funerary ritual reflects the central position of Pontecagnano in relation to the main territory of Etruria and long-range maritime traffic [72]. Between the 7th and 6th centuries a significant phenomenon of urbanisation took place, which was accompanied by the diffusion of the Etruscan culture [67]. Starting from the 5th century, Pontecagnano was influenced by the Samnites’ expansion, witnessed by the establishment of an Italic funerary ritual [73]. In the first half of the 3rd century, the Roman expansion ended the Etruscan-Samnite city [29].

The tombs here analysed are mainly from the Orientalizing period. The significant cultural and social changes of this period was reflected in new funerary practices and the abandonment of earlier necropolises [29]. Graves were organized in small plots, emphasizing generational ties, with more elaborate funerary goods influenced by foreign customs [70]. A distinctive feature in both adult and non-adult graves was the introduction of funerary vessels related to the consumption and ritual offering of wine, symbolizing privileged relationships with the Greek world and the East [27,28].

### Preparation of histological thin sections

Before sectioning, each tooth underwent thorough standard documentation [62]. High-resolution photographs were taken from multiple angles to preserve detailed records of each tooth. To preserve the crown surfaces for future studies, high-resolution replicas of the tooth crowns were produced [9,74]. All histological procedures were performed at the BIOANTH-Biological Anthropology and Dental Histology laboratory of Sapienza University of Rome.

For the obtainment of histological thin sections, the NOWA protocol by Esposito et al. [75] was followed. Teeth were first embedded in Crystalbond™ (Aremco Products, Inc.), and then in bicomponent epoxy resin (Buehler EpoxiCure™ 2). After curing, the resin blocks were sectioned using an IsoMet Low Speed Saw (Buehler). A longitudinal cut was made along the bucco-lingual plane passing next to the tip of the dentine horn, targeting the mesio-buccal cusp in molars [11,32]. The first cut produced two blocklets; the one with the dentine horn was selected for further processing, while the other half was stored for potential future analyses.

The selected blocklets were glued to microscope slides using a UV-adhesive (Loxeal® Engineering Adhesives UV 30–23) and cured under UV light. The mounted blocklets were sectioned using the diamond blade saw to produce thin sections of approximately 250–300 μm in thickness. Thin sections were polished using progressively finer abrasive papers (Carbimet Buehler - P800, P1200, P2500) to reduce thickness to an optimal range of approximately 80–100 μm for histological analysis [11]. Then, the sections were polished with alumina suspension (MicroPolish Alumina 0.3 μm, Buehler) and then mounted using Eukitt® mounting medium (O. Kindler GmbH & Co.) and covered with cover slips. Lastly, high-resolution photomosaics of the thin sections were acquired at different magnifications (50x, 100x) with polarized light using a transmitted light microscope (Zeiss Axio IMAGER.M2, Carl Zeiss Microscopy GmbH) equipped with a digital color camera for microscopy (Zeiss Axiocam 807, Carl Zeiss Microscopy GmbH). The photomosaics were automatically obtained via the “tiles” tool in ZEN Core (v3.8, Carl Zeiss Microscopy GmbH) software.

### Dental enamel histomorphometry

Chronologies of the dental crowns were constructed using the zig-zag method proposed by Dean [76]. This method involves marking prism segments and Retzius lines starting from the tip of the dentine horn up to the cervical area of the tooth. Data segments were traced and measured using ImageJ v.1.54p [77]. The measurements were then used to calculate the crown formation time (CFT) and the enamel extension rates (EER) using a standard daily secretion rate (DSR) of 2.85 µm/day [20]. The CFT represents the total time required for complete crown formation, calculated by summing the formation times of the measured prism segments along the crown [78–80]. The EER represents the rate of enamel formation along the EDJ, calculated by measuring the distance between prism segments and dividing it by the estimated time for each segment [20].

In first molars, chronologies were anchored to birth either through the identification of the Neonatal Line (NL), when present, or by aligning the start of crown formation with birth as per literature convention [80–82]. For worn first molars, the dentine horn tip was digitally reconstructed using an overlay of a well-preserved tooth. The EDJ path was traced, and additional days of enamel formation were added based on the length of the reconstructed segment, providing a calibrated starting point for the chronology.

**Fig 3:**
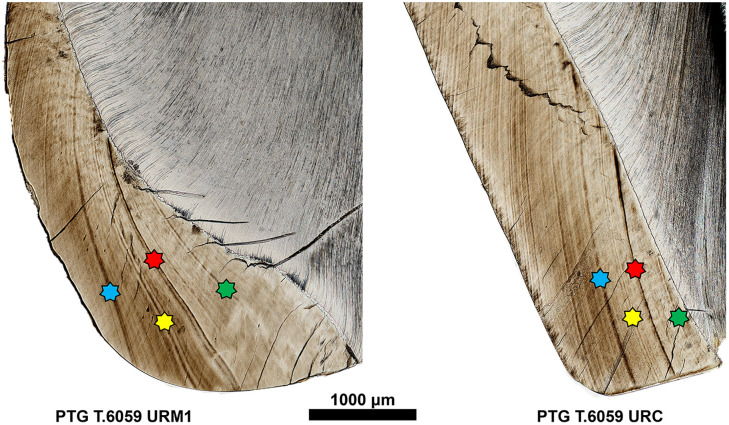
Photomosaics of URM1 and URC teeth from individual PTG T.6059 with matched Accentuated Lines (ALs). Colors of the star symbols indicate homologous ALs, matched by morphology and spacing along the enamel, enabling alignment of developmental chronologies between teeth.

ALs were identified if they were visible for at least three-quarters of their length, as suggested by Nava et al. [3], and if they were observed, at least partially, on both the labial and buccal sides of the crown [4,37,83]. The dental crowns of each individual were aligned by registering sequences of ALs (Fig 3), used as temporal markers. The methodology is based on the hypothesis that AL patterns in enamel (morphology and distance) are maintained across the different teeth forming at the same time [3,4].

The prevalence of stresses was calculated as the number of individuals with at least one AL in a specific month from birth, divided by the number of individuals with enamel in formation during that same month, and expressed as a percentage [3,4]. Statistical analyses and graphs were performed using R version 4.5.1 [84].

### Dental calculus

Dental calculus sampling was conducted following the protocol systematized by Sabin and Fellows Yates [85], with some variations (e.g., disposable blades were changed after each sample extraction). Decontamination procedures, demineralization and optical analyses followed standard published protocols [5,51,85,86] and were conducted in dedicated clean spaces not connected to modern botanical work and under strict environmental monitoring at the DANTE-Diet and Ancient Technology laboratory of Sapienza University of Rome.

Calculus samples were cleaned in order to remove soil particles and other potential environmental contaminants attached to their surface. Bench surfaces were cleaned throughout the multiple steps of the sample preparation, using soap and ethanol, and starch-free nitrile gloves were used at all times. Each sample was decontaminated under a stereomicroscope, on a clean Petri dish, with magnifications of up to 100x. During this procedure, the removal of soil adhering to the surface of the calculus was performed using sterile tweezers to hold the sample and a fine acupuncture needle to gently scrape off the soil attached to the external layer of the mineralized plaque. Drops of 0.5 M HCl acid were used to dissolve the flecks of soil and ultrapure water to halt demineralization, as well as to wash and remove contaminants. The decontamination water was stored in plastic tubes for monitoring purposes. The clean calculi were then washed in ultrapure water up to three times to remove any traces of loose sediment, dissolved in a solution of 0.5 M HCl, and subsequently mounted on slides using a solution of 50:50 glycerol and ultrapure water. Furthermore, environmental samples from dust-traps set in the laboratory were collected in order to rule out any type of modern contamination.

Glass slides were analyzed using a Zeiss Axio Imager.M2 with magnifications ranging from 100x to 630x. A modern reference collection of 300 plants native to the Mediterranean region and Europe, along with published literature, was used for the identification of archaeological starch granules. Spores were identified by comparison with available literature [87,88].

## Results

### Histomorphometry results

CFTs for each tooth were estimated through histomorphometric analysis and are reported in Table 2 and S1 Table, alongside the initial cusp formation (Ci) and crown completion (Crc) rates over the individual’s lifetime. These rates are expressed in days, months, and years of life. Specifically, canine CFTs range from approximately 1,560 days (4.3 years) to 2,498 days (6.8 years). In contrast, first molars have a shorter CFT, ranging from 860 days (2.4 years) to 1,304 days (3.6 years), while second molars have CFTs ranging from 896 days (2.5 years) to 1,428 days (3.9 years). Regarding Ci, it is important to note that, when the NL was not visible, the apex of the dentin horn tip was considered day 0 for first molars. For canines and second molars, however, overlapping ALs are used for calibration, although some of these are still worn. It was not possible to overlay only the M2 of individual PTG T.5980, so three years were given as Ci in this case, as indicated in the literature [80].

**Table 2:** Crown Formation Time (CFT), Initial Cusp Formation (Ci), and Crown Completion (Crc) values expressed in days, months, and years for each individual and tooth class.

ID sample	ID tooth	CFT days	CFT months	CFT years	Ci days	Ci months	Ci years	Crc days	Crc months	Crc years
	LLP4	>1231	>40.4	>3.4	>890	>29.2	>2.4	>2121	>69.7	>5.8
PTG T.5980	LLM1	860*	28.3*	2.4*	0.0	0.0	0.0	860	28.3	2.4
	LLM2	896	29.4	2.5	1096	36.0	3.0	1992	65.4	5.5
	ULC	>2392	>78.6	>6.5	>184	>6.0	>0.5	>2576	>84.6	>7.1
PTG T.5998	ULM1	1149*	37.8*	3.1*	0	0.0	0.0	1149	37.8	3.1
	ULM2	1363	44.8	3.7	1179	38.7	3.2	2542	83.5	7.0
	LLC	>2045	>67.2	>5.6	>230	>7.5	>0.6	>2275	>74.7	>6.2
PTG T.6036	ULM1	1073*	35.2*	2.9*	−62	−2.1	−0.2	1010	33.2	2.8
	URM2	1154	37.9	3.2	1018	33.4	2.8	2172	71.4	5.9
	URC	>1823	>59.9	>5.0	>173	>5.7	>0.5	>1996	>65.6	>5.5
PTG T.6059	URM1	960*	31.5*	2.6*	0	0.0	0.0	960	31.5	2.6
	URM2	1130	37.1	3.1	1113	36.6	3.0	2243	73.7	6.1
	URC	>1915	>62.9	>5.2	>70	>2.3	>0.2	>1984	>65.2	>5.4
PTG T.6073	URM1	1034*	34.0*	2.8*	0	0.0	0.0	1034	34.0	2.8
	LLM2	1240	40.7	3.4	1011	33.2	2.8	2251	74.0	6.2
	URC	>1824	>59.9	>5.0	>125	>4.1	>0.3	>1949	>64.0	>5.3
PTG T.6921	URM1	908	29.8	2.5	0	0.0	0.0	908	29.8	2.5
	URM2	913	30.0	2.5	1044	34.3	2.9	1957	64.3	5.4
	LRC	>2498	>82.1	>6.8	>121	>4.0	>0.3	>2619	>86.0	>7.2
PTG T.8356	LLM1	1304*	42.8*	3.6*	0	0.0	0.0	1304	42.8	3.6
	LRM2	>1269	>41.7	>3.5	>1733	>56.9	>4.7	>3002	>98.6	>8.2
	ULC	>1855	>61.0	>5.1	>269	>8.8	>0.7	>2125	>69.8	>5.8
PTG T.8360	LLM1	1250*	41.1*	3.4*	0	0.0	0.0	1250	41.1	3.4
	LLM2	1294	42.5	3.5	>983	>32.3	>2.7	>2276	>74.8	>6.2
	URC	1560	51.3	4.3	97	3.2	0.3	1658	54.5	4.5
PTG T.8390	LLM1	1141	37.5	3.1	−23	−0.7	−0.1	1118	36.7	3.1
	LLM2	1068	35.1	2.9	1182	38.8	3.2	2250	73.9	6.2
	LLC	>1885	>61.9	>5.2	>187	>6.1	>0.5	>2071	>68.0	>5.7
PTG T.8428	LRM1	1265*	41.6*	3.5*	0	0.0	0.0	1265	41.6	3.5
	LRM2	1428	46.9	3.9	1218	40.0	3.3	2646	86.9	7.2

The symbol ‘>’ indicates cases where the dentin horn tip is slightly worn, meaning the actual value may be greater than the calculated value. The symbol ‘*’ denotes values derived from a group-specific model reconstruction (i.e., reconstruction of the dentin horn and the first prism segment in the enamel).

Table 3 shows the mean CFTs, Ci and Crc, and their respective standard deviations for each tooth class (excluding the single P4). On average, canines take about 1,977 days (5.4 years) to form, while first molars take about 1,094 days (3.0 years) and second molars take about 1,176 days (3.2 years). These values highlight the variation in CFT between tooth classes, with canines exhibiting significantly longer CFTs than the first and second molars. The average Ci for the first molars is shifted by a few days before birth (−8 days), as the NL was visible in two permanent first molars. Canines show an average formation onset of 162 days, while second molars show an average of 1,158 days. In contrast, the Crc mean values are 1,086 days for M1s, 2,139 days for Cs, and 2,333 days for M2s.

**Table 3:** Mean and standard deviations (SD) of Crown Formation Time (CFT), Initial Cusp Formation (Ci), and Crown Completion (Crc) across different tooth classes.

Tooth class	CFT days mean	CFT days SD	Ci days mean	Ci days SD	Crc days mean	Crc days SD
**C**	1977	295	162	64	2139	307
**M1**	1094	154	−8	20	1086	156
**M2**	1176	179	1158	218	2333	316

Negative Ci values indicate crown formation initiation before birth.

**Fig 4:**
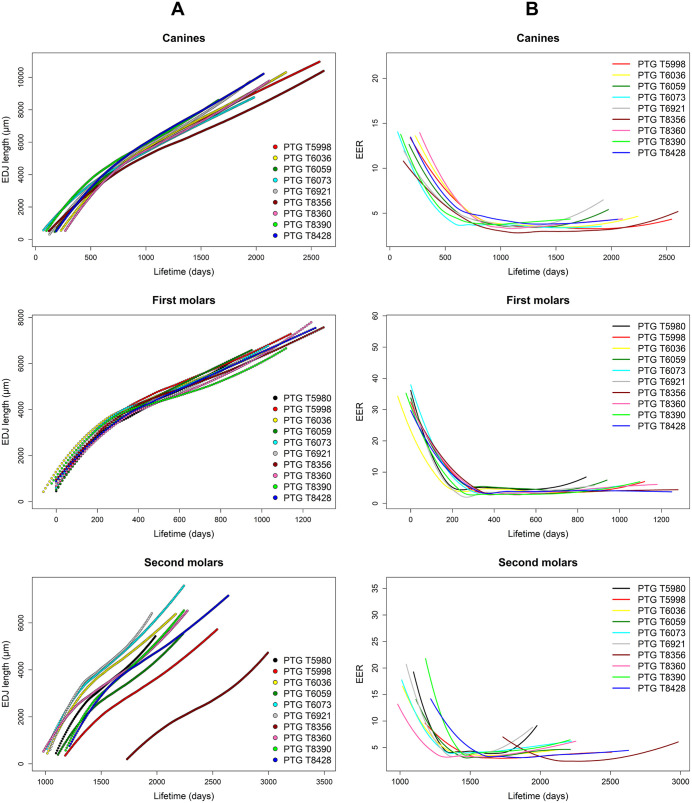
Enamel-dentine junction (EDJ) growth and enamel extension rate (EER) variation across crown formation days. Growth curves of dental crowns along the enamel-dentine junction (EDJ) over crown formation days **(A)** and enamel extension rate (EER) variation over crown formation days **(B)**. The graphs, divided by tooth class, illustrate crown growth dynamics across different individuals, showing EDJ length progression and changes in EER values over time.

Fig 4A presents the growth curves of dental crowns, showing cumulative length along the EDJ as a function of crown formation days. M1 and M2 crowns exhibit rapid growth during the first 200 days, followed by a gradual deceleration toward the cervical region. C crowns undergo an accelerated growth phase during the first 400 days before slowing progressively. Fig 4B illustrates the variation in EER values relative to crown formation days. Initial EER values are significantly higher than subsequent ones, indicating a faster ameloblast recruitment rate in the cusp region, which progressively slows as it approaches the cervical area. The deceleration in EER values along the EDJ is consistent across all tooth classes. As shown in the M2 graphs, the curve of individual PTG T.8356 is shifted forward in time, reflecting a delayed onset and completion of crown formation (Ci and Crc) compared to the other individuals in the sample.

Table 4 and S1 Table report respectively the number and the chronology of ALs for each tooth and individual. The average of stress events during childhood is 25.7 (SD 8.7), with homologous ALs across different teeth counted as a single stress event. Notably, canines consistently recorded the highest number of ALs among all dental classes.

**Table 4:** Number of Accentuated Lines (ALs) detected in each tooth and individual.

ID sample	ALs M1	ALs C	ALs M2	ALs Tot
PTG T.5980	9	10	5	19
PTG T.5998	7	14	5	17
PTG T.6036	8	18	12	28
PTG T.6059	9	12	8	22
PTG T.6073	19	26	11	42
PTG T.6921	9	16	11	30
PTG T.8356	11	20	5	25
PTG T.8360	19	25	10	36
PTG T.8390	7	12	12	19
PTG T.8428	9	12	7	19
Mean	10.7	17.2	8.6	25.7
SD	4.5	5.5	3.0	8.3

The table reports AL counts for first molars (M1), canines (C), and second molars (M2). It also shows the total number of ALs per individual. Mean values and standard deviations (SD) are provided for each tooth class and the overall totals.

**Fig 5:**
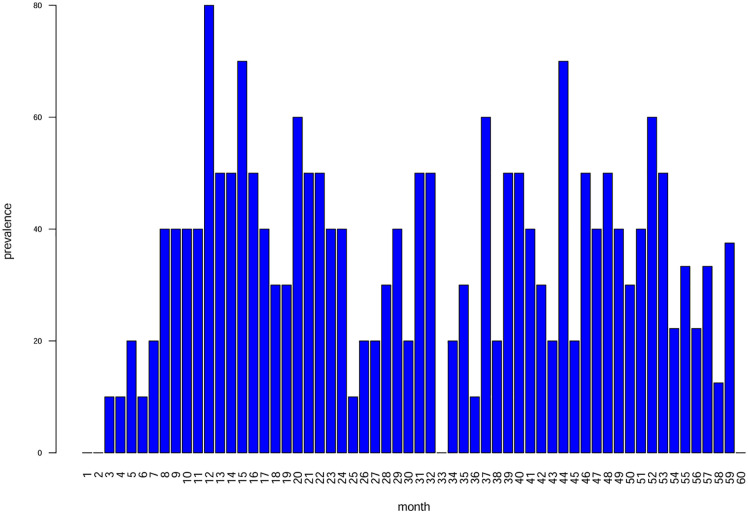
Graph showing the monthly prevalence of Accentuated Lines (ALs) observed in the sample. The x-axis represents age in months, while the y-axis indicates prevalence percentage. Peaks at specific ages suggest potential physiological stress events during childhood.

The monthly prevalence of stress events – calculated as the proportion of individuals presenting at least one AL within a given month, relative to the number of individuals with teeth forming during that period – is shown in Fig 5. The distribution exhibits an asymmetric pattern: prevalence is null during the first two months of life, after which it starts to increase; from the seventh month onwards, prevalence drastically increases, reaching its peak at the twelfth month, where 80% of the sample presents at least one AL. This produces a bell-shaped distribution centered on the first year of life. Subsequent months show fluctuating values, with a second peak observed around the forty-fourth month. From the sixtieth month, a progressive decline in both the rate and denominator of prevalence values is observed, and the graph was therefore cut at this month.

**Fig 6:**
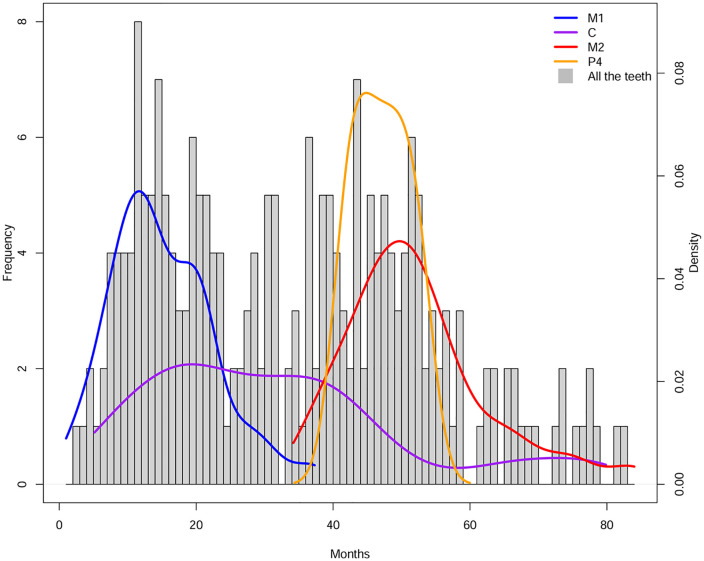
Density curves illustrate the distribution of Accentuated Lines (ALs) across different tooth classes. The x-axis represents age in months, while the y-axis shows frequency (left) and density (right). The curves for first molars (M1), canines (C), second molars (M2), and premolar (P4) highlight variations in stress occurrence throughout childhood.

Differential analysis of stress occurrence across dental classes is presented in Fig 6. Density plots of ALs distribution throughout the crown indicate that both the cuspal (initial) and cervical (terminal) regions exhibit fewer stress markers compared to the lateral (central) crown portion, which displays the highest concentration of ALs.

### Calculus analysis results

The dental calculus preserved vegetal micro-remains in the form of starch granules, plant fibers, and fungal spores (Table 5).

**Table 5:** Overview of plant and fungal micro-remains detected in dental calculus analysis.

ID	Starch (Triticeae)	Starch (Fabaceae)	Starch indefinite	Fungal spores	Plant fibers
**PTG T.5998**	1	1		numerous	numerous
**PTG T.8340**	1				numerous
**PTG T.8356**				numerous	1
**PTG T.8360**				numerous	numerous
**PTG T.8428**	1	1	1		numerous

The presence of micro-remains is indicated numerically, where ‘1’ represents a single occurrence, and ‘numerous’ denotes abundant traces of the same type.

**Fig 7:**
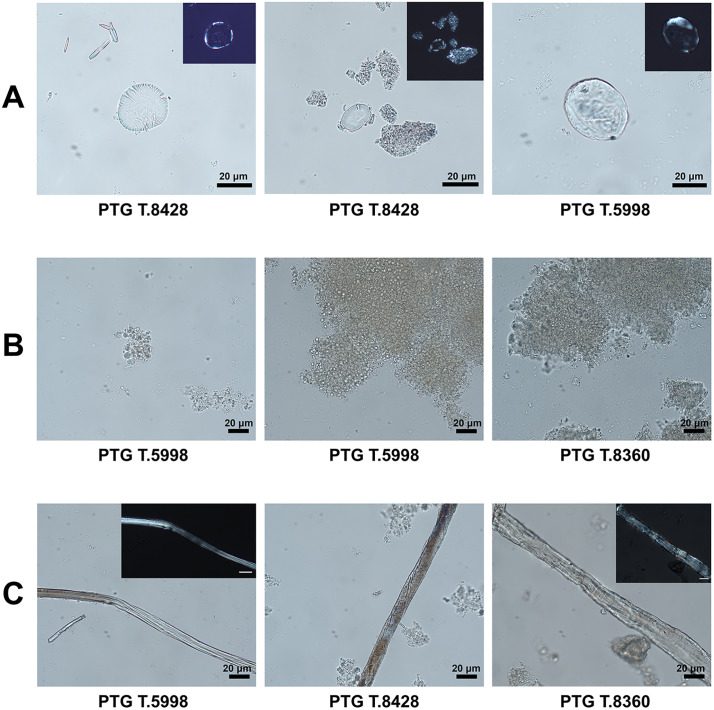
Micro-remains extracted from dental calculus, observed under optical microscopy. The images show different types of residues: **(A)** starch granules, suggesting the consumption of plant-based foods; **(B)** fungal spores of *Saccharomyces*, possibly associated with the intake of fermented foods or beverages; **(C)** plant fibers, which may reflect para-masticatory activities.

Starch granules (Fig 7A) were recovered in three of the five individuals analyzed. Overall, two morphotypes were identified in this study. To avoid misinterpretation, starch granules smaller than 5 μm (transient starches) were excluded from the analysis [89].

*Morphotype I*: Starch granules of this type were found in the dental calculus samples of three individuals (PTG T.8340, PTG T.8428, PTG T.5998), with some still partially embedded within the calculus matrix. The morphotype is typically characterized by large granules (sub-type A), which displays a round to sub-oval two-dimensional shape, a centric hilum, and well-defined lamellae, with maximum widths ranging from 21.1 and 45.1 μm (mean: 33.1 μm). Under polarized light, a centric extinction cross with four arms is visible. In both experimental and published reference collections, starch granules of this morphotype are typically found in bimodal distributions, with large sub-type A granules associated with smaller granules (sub-type B), usually < 10 μm, also round/sub-oval in shape and with a central hilum [90–92]. However, in archaeological samples, sub-type B granules are often underrepresented or absent, as in this case, likely due to differential preservation. According to the literature and modern reference collections, the observed morphometric features – along with bimodal distribution when preserved – are consistent with starch granules from members of the Triticeae tribe (Poaceae family) and are considered diagnostic for taxonomic identification [54,91,92].

*Morphotype II*: A single starch granule of this type was identified in two individuals (PTG T.8428, PTG T.5998). These granules exhibit a reniform shape, with size ranging between 12–35 μm, and a collapsed/sunken hilum forming a deep fissure extending along almost the entire granule. Under polarized light, a bright extinction cross was visible with several lateral arms (> 4) diverging from the centric hilum [93]. These features are diagnostic of starch granules in the Fabaceae family, known for several edible domesticated legumes (e.g., *Lens culinaris* Medikus, *Vicia faba* L., and *Pisum sativum* L.) and wild vetches (*Vicia* spp.). Identification at species or genus level was not possible due to overlaps in shape and size at tribe level, which were observed in our modern reference collection.

Several *Saccharomyces* fungal spores (Fig 7B) were identified in three individuals (PTG T.8356, PTG T.8360, PTG T.5998). Known as “sugar fungi,” they include several yeasts [94,95].

Plant fibers and wood elements (Fig 7C) were identified in the calculus of all the analyzed individuals. The fibers are long and, in some cases, were observed still embedded in the mineral matrix. Several display diagnostic morphological features – including distinct longitudinal striations, thickened cell walls, and occasional Z-twisting – clearly visible under polarized light. These characteristics are consistent with bast fibers derived from plants such as *Linum usitatissimum* or *Cannabis sativa* and align with established identification criteria [96,97]. The presence of these fibers may reflect exposure to fibrous plant materials through extra-masticatory or occupational activities, such as fiber processing, cordage production, or the use of plant implements for oral hygiene.

## Discussion

This study represents an approach to biological life histories reconstruction using dental histomorphometry and calculus micro-residue analysis on a sample of permanent teeth from the Pontecagnano community (7th-6th century BCE). This interdisciplinary approach allowed the reconstruction of aspects of individual life histories, including growth trajectories and health conditions, and provides site-specific insights into lifestyle during the Iron Age. While overall lifespan is often regarded as a direct proxy for human health and environmental adaptation, the frequency and timing of non-fatal stress events, such as illnesses or nutritional deficiencies, throughout development can provide significant insights into a community’s adaptive capacity and biological success [98].

The CFTs obtained in this study reveal significant differences between tooth classes (Tables 2 and 3), with canines showing the longest formation times (mean = 1,977 days, SD = 295 days, N = 9), followed by second molars (mean = 1,176 days, SD = 179 days, N = 10) and first molars (mean = 1,094 days, SD = 154 days, N = 10). These results are in line with Reid and Dean [80], who documented similar trends in enamel formation time in modern human teeth, highlighting the prolonged development of anterior teeth compared to molars. Regarding Ci, our results show that the mesio-buccal cusp of the first permanent molars begins formation shortly before birth (mean = −8 days, SD = 20 days). However, this estimate should be interpreted with caution: of the ten M1s analyzed, the Neonatal Line (NL) was clearly visible in only two cases – specifically, in the mesio-buccal cusp of the LLM1 of individual PTG T.8390 and in the mesio-buccal cusp of the ULM1 of individual PTG T.6036. In all other cases, crown initiation was anchored to birth according to standard conventions in the absence of a visible NL [80–82]. Canines initiate crown formation a few months later (mean = 162 days, SD = 64 days), while second molars begin considerably later (mean = 1,158 days, SD = 218 days), as detailed in Table 3. Reid and Dean [80] report fixed values for Ci, with M1s always forming at birth, M2s at 3 years of age, and canines at 274 days (for upper) and 200 days (for lower). Our Ci values for M1 and M2 are generally consistent with these estimates, while Ci values for C are generally lower. Regarding Crc, our results indicate that crowns of M1s complete at an average of 1,086 days (SD = 156 days), those of canines at 2,139 days (SD = 307 days), and those of M2s at 2,333 days (SD = 316 days). Notably, the M2 of individual T.8356 has extreme Ci and Crc values. In fact, this M2 begins forming around 1.5 years later than the others and consequently finishes forming later. However, these values fall within the expected range of human variability in enamel formation timing [65] and likely reflect inter-individual developmental differences. Unlike previous studies that rely on literature-based Ci values – resulting in Crc estimates derived from fixed starting points – our approach allows for greater precision. The Ci values for C and M2 were determined through chronological overlap with M1, ensuring more accurate estimations of crown completion. Furthermore, the general trends observed in our study are consistent with data obtained by AlQahtani et al. [65] on enamel formation rates among tooth classes.

Differences in EERs were observed across tooth classes (Fig 4). M1 and M2 crowns exhibited a rapid initial extension during the first 200 days, followed by a gradual deceleration toward the cervical region. C crowns showed a prolonged phase of accelerated growth, lasting up to 400 days before slowing down. These findings align closely with those of Guatelli-Steinberg et al. [20], who reported an exponential decline in EER from the cusp to the cervical region in modern human teeth. Both studies highlight that the highest EER values occur in the cuspal region, gradually decreasing as enamel formation progresses cervically. These differences emphasize the variability in EERs among different tooth types, which can be attributed to distinct morphological and developmental characteristics. The observed trend in EER values over time follows a general decrease in enamel formation rates as crown development advances.

There is a lack of comparative data on histomorphometric parameters from the Iron Age in the literature, which refers to different chronological and geographical periods [11,99]. Both Nava et al. [11] and Aris et al. [99] report a diachronic trend of slowing childhood growth trajectories, even in relatively recent evolutionary times. However, only Aris et al. [99] consider the permanent dentition. This study provides, for the first time, histological data from an Iron Age Italian community.

In this study, the prevalence of ALs provided a longitudinal picture of health conditions from birth up to ca. 6 years of age. The monthly distribution of ALs (Fig 5) shows an asymmetrical, almost bimodal, distribution with notable peaks at 12 and 44 months from birth. These non-fatal stress events are typically linked to infectious diseases and nutritional deficiencies [12,37,38,100,101]. The lack of stress events in the first two months of life aligns with maternal antibody protection [102], while later peaks may be related to increased exposure to pathogens and/or dietary changes. The initial rise in stress prevalence, observed from the third month, may coincide with infants becoming more physically engaged with their surroundings – sitting up, touching objects, and experiencing increased exposure to environmental pathogens [103].

Differential analysis of stress registration across the tooth crowns (Fig 6) indicates that both the cuspal and cervical regions record stress less effectively compared with the central portion of the crown. If the entire crown were equally susceptible, a homogeneous distribution of stress markers would be expected; instead, the observed bell-shaped pattern underscores the differential recording susceptibility across the crown. Thus, examining multiple tooth types enhances the likelihood of capturing stress events that might otherwise go undetected in a single dental class. This observation confirms previous findings by Nava et al. [3], who identified similar crown regional trends in both deciduous and permanent teeth. It is important to note that, during the first months of life, only M1s are forming enamel, and exclusively in their cuspal regions, which are less susceptible to stress recording. This may help explain the initial absence of stress markers observed in the enamel, and consequently in the prevalence curve, during the first two months. Conversely, the second peak around 44 months likely reflects stress signals recorded mainly by M2s, whose enamel at that stage is forming in the central crown regions – areas shown to register stress more effectively.

**Fig 8:**
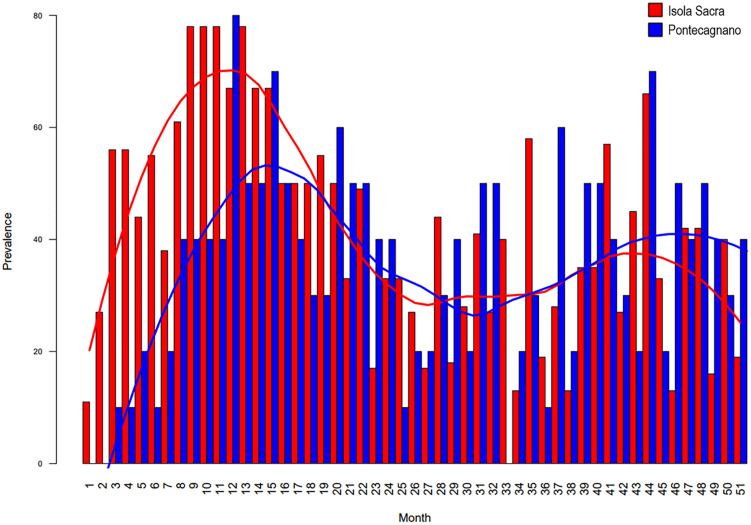
Comparison of monthly stress prevalence between Pontecagnano (this study) and Isola Sacra [3]. The graph illustrates variations in stress prevalence over time, with the x-axis representing age in months and the y-axis indicating prevalence percentage. Color-coded bars and trend lines highlight differences and similarities between the two populations.

The ALs prevalence distribution partly aligns with a similar study presented by Nava et al. [3] in a Roman Imperial sample from Isola Sacra (Fig 8). Nava et al. [3] interpreted the observed ALs prevalence distribution in their sample as indicative of weaning-related stress, with the highest frequency of stress events occurring around the first year of life. In our Pontecagnano sample, however, the primary peak is slightly shifted forward at approximately 12 months. This difference may be explained by the differential recording capacity within the dental crown. As demonstrated by Nava et al. [3], deciduous teeth can record stress events during the first months of life – a stage when the crowns of deciduous canines and second molars are midway through their development. At this point, enamel is forming in the central regions, which are particularly sensitive to physiological stress and well suited for its detection. In contrast, in our sample, stress in the perinatal period can only be registered by M1s, which at that stage are forming enamel only in the cuspal region – an area less responsive to stress recording. Consequently, the absence of deciduous teeth in our dataset possibly shifts the overall ALs prevalence profile forward, potentially leading to a slight underestimation of early-life stress events.

A more challenging interpretation concerns the secondary rise in the AL prevalence curve, with a peak around 44 months. Nava et al. [3] acknowledge that, as children become more mobile and interact more actively with their surroundings, increased exposure to pathogens may contribute to further stress events. In our sample, it is also important to note that this second peak is very likely explained by the fact that, at this stage, enamel is forming almost exclusively in the central regions of M2s – areas that are highly responsive to stress recording. Additionally, this peak may coincide with weaning completion and the transition to a diet more closely resembling that of adults, reflecting the metabolic challenges associated with the diversification of food sources, both of which may pose significant physiological stress during early childhood. Given the non-specific nature of ALs as stress markers, it is plausible that both increased environmental exposure and the dietary transition toward an adult-like pattern contribute to this more complex stress profile.

Overall, the ALs prevalence distribution in our Pontecagnano sample reflects the short-term, non-fatal stresses that individuals experienced during the first 6 years of life. It is important to highlight that although the series considered in this analysis is essentially a mortality sample, the selected individuals were adults who passed away long after the complete formation of their dental crowns. Therefore, all individuals survived the stressful events that characterized their childhood.

During the Orientalizing period, the Etruscan civilization experienced significant socio-economic and cultural changes, characterized by demographic growth, an increase in agricultural activity, and the emergence of a stratified society [70]. This period also saw the expansion of Etruscan culture across various areas of central Italy, with a notable expansion into Campania [104,105]. The Villanovan and Etruscan diet was predominantly plant-based, with a moderate intake of animal proteins, as demonstrated by isotope analysis [106,107] and archaeobotanical and zooarchaeological studies [108–110]. Nutrition was primarily based on cereals such as emmer spelt, durum wheat, barley, and rye [111], while legumes – including beans, chickpeas, lentils, lupins, and peas – complemented the diet by providing an alternative source of proteins [112]. Animal food sources included sheep, goats, cattle, wild boars, and small game [113].

Dental calculus analysis of Pontecagnano individuals revealed the presence of starch granules, fungal spores, and plant fibers (Fig 7), which provide complementary insights into both adult diet and non-dietary activities. Starch granules from the Triticeae (Poaceae) and Fabaceae families were detected in three individuals, indicating the consumption of cereals and legumes in line with diversified agricultural practices already documented for ancient Italian populations [110,114]. The damaged appearance of some starch granules could suggest food processing activities such as cooking and/or seed grinding, as well as enzymatic degradations resulting from early stages of starch digestion prior to consumption. In addition, the presence of oral diseases such as caries, documented in several individuals from the Pontecagnano sample, is consistent with a regular intake of carbohydrates, a pattern already well documented in agricultural populations since the Neolithic [57,115,116]. Interestingly, numerous *Saccharomyces* spores were identified in three individuals (two males and one of unknown sex). Their abundance in the dental calculus sample analyzed may indicate regular consumption of fermented foods or beverages – a practice already attested in archaeological contexts through the analysis of the mineralized matrix, primarily via proteomic methods and, more rarely, through optical microscopy as in our study [55,117]. These findings can be contextualized within the broader socio-cultural transformations of the Orientalizing period at Pontecagnano, when agricultural intensification and increasing contacts with the Mediterranean world contributed to shaping resource availability and dietary habits. Finally, the presence of plant fibers in all individuals suggests possible extra-masticatory use of the teeth. In several individuals, long and fragmented fibers were still embedded in dental calculus, some exhibiting characteristics indicative of cortical fibers. The widespread presence of wood particles in the sample suggests inhalation associated with extra-masticatory activities – many of which may be occupational in nature – or the use of wooden implements related to food preparation or oral hygiene within the Pontecagnano community. As commonly observed in dental calculus studies, such inclusions may also derive from other environmental or occupational pollutants [5].

All in all, the variety of microremains recovered from the Pontecagnano individuals highlights the advantages of dental calculus analysis for reconstructing ancient diet and lifeways. Thanks to its mineralized structure, dental calculus can preserve starches, phytoliths, plant remains, and biomolecules such as DNA and proteins, providing unique direct evidence of plant food consumption that is often inaccessible through more protein-sensitive methods like traditional isotopic analysis. Furthermore, the potential taxonomic identification of starch granules can reveal the photosynthetic pathways of plants (C_3_ vs. C_4_), offering further insights into the relative contribution of different plant resources to ancient diets. Given the small number of individuals with preserved dental calculus, the results of this analysis should be interpreted with caution and are best understood as providing detailed insights into individual life histories, rather than as representative of the broader population.

Although reconstructing infant feeding practices in past populations is inherently challenging due to the scarcity of direct evidence, our study adds new data to the debate, particularly regarding early-life dietary stress and the physiological impact of weaning. A recent isotopic study on individuals from Pontecagnano [107] during the Orientalizing period suggests a diet primarily based on C_3_ plants, with limited intake of animal proteins and isolated evidence of C_4_ plant consumption – possibly millet [107]. According to the WARN model applied in that study, weaning timing ranged between 0.7 years (t_1_, onset of weaning) and 2.6 years (t_2_, end of weaning) [107]. The reported onset of weaning align with the increase in physiological stress markers observed in our sample, and the presence of C_3_ plant consumption align with our analysis of dental calculus micro-remains.

## Conclusions

This study, the first one to apply dental histology analysis to a sample from the Pontecagnano necropolises, does not aim to provide population-wide generalizations, but rather to offer detailed insights into individual life histories and site-specific patterns. The unique integration of dental calculus analysis and dental histomorphometry enabled us to investigate dietary and behavioral aspects in Pontecagnano adult individuals through the micro-residues incorporated in dental calculus and, at the same time, explore growth trajectories and health profile – derived from enamel histomorphometry – during the same individuals’ infancy and childhood. The pseudo-longitudinal histological methodology applied to adult permanent teeth has proven effective in capturing the temporal dynamics of stress during infancy and early childhood and has also provided important insights into developmental timing. In parallel, dental calculus analysis has provided valuable data on adults’ diet and para‐masticatory activities, revealing evidence for cereal and legume consumption as well as indications of food processing and fermentation practices. Overall, the integrated use of these techniques offers a detailed representation of childhood development and its links with adult biocultural practices. Future research should integrate additional analytical methods – such as dental microwear studies [9,118], which provide evidence of food mechanical properties and texture, and high-resolution laser-based *in situ* trace element and isotopic analyses on dental enamel [1], which shed light on dietary composition, weaning practices, and mobility – on a larger sample of the ancient community of Pontecagnano.

## Supporting information

S1 Table Histomorphometric data of all sampled individuals. (XLSX)
